# Evolution of host plant use and diversification in a species complex of parasitic weevils (Coleoptera: Curculionidae)

**DOI:** 10.7717/peerj.6625

**Published:** 2019-03-20

**Authors:** Gerardo Hernández-Vera, Ivo Toševski, Roberto Caldara, Brent C. Emerson

**Affiliations:** 1 School of Biological Sciences, University of East Anglia, Norwich, Norfolk, UK; 2 Instituto de Botánica, Departamento de Botánica y Zoología. Centro Universitario de Ciencias Biológicas y Agropecuarias, Universidad de Guadalajara, Zapopan, Jalisco, México; 3 CABI Switzerland, Delémont, Switzerland; 4 Department of Plant Pests, Institute for Plant Protection and Environment, Zemun, Serbia; 5 Center of Alpine Entomology, University of Milan, Milan, Italy; 6 Island Ecology and Evolution Research Group, IPNA-CSIC, La Laguna, Tenerife, Canary Islands, Spain

**Keywords:** Curculionidae, Host plant use, Insect-plant interactions, Diversification, Evolutionary ecology

## Abstract

Weevils (Coleoptera: Curculionoidea) represent one of the most diverse groups of organisms on Earth; interactions with their host plants have been recognized to play a central role in their remarkable diversity, yet the exact mechanisms and factors still remain poorly understood. Using phylogenetic comparative analyses, here we investigate the evolution of host use and its possible role in diversification processes of *Rhinusa* and *Gymnetron*, two closely related groups of weevils that feed and develop inside plant tissues of hosts within the families Scrophulariaceae and Plantaginaceae. We found strong evidence for phylogenetic conservatism of host use at the plant family level, most likely due to substantial differences in the chemical composition of hosts, reducing the probability of shifts between host families. In contrast, the use of different plant organs represents a more labile ecological trait and ecological niche expansion that allows a finer partitioning of resources. *Rhinusa* and *Gymnetron* weevils initially specialized on plants within Scrophulariaceae and then shifted to the closely related Plantaginaceae; likewise, a gall inducing behavior evolved from non-galler weevils, possibly in response to resource competition, as galls facilitate larval development by providing enhanced nutrition and a favorable microhabitat. Results from trait-dependent diversification analyses suggest that both use of hosts within Plantaginaceae and parasitism on fruits and seed capsules are associated with enhanced diversification of *Rhinusa* and *Gymnetron* via low extinction rates. Our study provides quantitative evidence and insights on the ecological factors that can promote diversification in phytophagous insects that feed and develop inside plant tissues.

## Introduction

Comprising approximately 5,800 genera and more than 60,000 described species (Oberprieler, Anderson & Marvaldi, 2014; Oberprieler, Marvaldi & Anderson, 2007), weevils (Coleoptera: Curculionoidea) have been described as one of the most successful adaptive radiations on Earth (Mayr, 1963; McKenna et al., 2009). The evolution of a rostrum, shifts in larval feeding habits and co-evolutionary relationships with flowering plants have been proposed as likely explanations for their great diversity (Marvaldi et al., 2002; McKenna et al., 2009; Oberprieler, Marvaldi & Anderson, 2007); however, the factors and processes involved are still little understood. Using every plant part and nearly every plant taxon (Anderson, 1995; McKenna et al., 2009), weevils often exhibit a close relationship with their host plants and, as is frequently observed in many other plant-feeding insect groups (Futuyma, Keese & Scheffer, 1993; Jaenike, 1990; Janz & Nylin, 1998), specialization on one or a few closely related host plant species is a recurrent phenomenon (Marvaldi et al., 2002). In some cases life history attributes such as endoparasitism, in which larvae not only feed but also develop inside a great variety of plant structures, contribute to a more intimate host association, which in turn may amplify the selection pressure imposed by the host, making weevils more susceptible to ecological divergence (Hernández-Vera et al., 2010; Mopper, 1996).

Here, we focus attention on species from the closely related genera *Rhinusa* and *Gymnetron* (Curculionidae: Curculioninae), endophagous parasitic weevils whose larvae feed and develop within tissues of plant species in the families Scrophulariaceae and Plantaginaceae. The genus *Rhinusa* comprises approximately 40 species with a Palearctic distribution (Caldara, 2001, 2013) that feed on species within the plant genera *Verbascum* and *Scrophularia* in the family Scrophulariaceae and *Linaria*, *Kickxia*, *Chaenorhinum*, *Antirrhinum* and *Misopates* within the family Plantaginaceae (Caldara, Sassi & Toševski, 2010). *Gymnetron* includes approximately 30 species with a Palearctic distribution (Caldara, 2008) and approximately 60 species from the Afrotropical region, of which 55 are known mainly from South Africa and considered to be endemic to this area (Caldara, 2003). All Palearctic *Gymnetron* use species from the genus *Veronica* (Plantaginaceae) as host plants (Caldara, 2008), whereas representatives from the Afrotropical region use different host genera within the plant family Scrophulariaceae, namely, *Hebenstreitia*, *Sutera*, *Selago*, *Pseudoselago*, *Tetraselago*, *Buddleja*, *Diascia*, *Nemesia*, *Hemimeris* and the genus *Anastrebe* in the family Stilbaceae (Caldara, 2003; Caldara, Colonnelli & Osella, 2008, 2009). At the intraspecific level, mitochondrial and nuclear DNA sequence data has revealed cryptic host-associated diversity within the species *Rhinusa antirrhini* (Hernández-Vera et al., 2010) and *R. pilosa* (Toševski et al., 2015); several evolutionary lineages were found to be associated with species and subspecies in the genus *Linaria*, suggesting host specialization as a likely driver for diversification.

Caldara, Sassi & Toševski (2010) suggest that *Rhinusa* species typically exhibit host conservatism at the plant family level and perhaps at the plant genus level for some species. Similarly, *Gymnetron* species appear to show host conservatism at the plant family level (Caldara, 2008; Caldara, Colonnelli & Osella, 2008); however, the extent to which host plant use is phylogenetically conserved across species within both genera remains to be explored quantitatively. Furthermore, given the intimate association of larvae with plant organs such as fruits, stems, roots and induced galls (Caldara, 2001; Toševski, Gassmann & Desančić, 2007), this group of weevils provides an excellent opportunity to test for phylogenetic conservatism in both associations with different host plants and larval parasitic modes. Within Coleoptera, larval feeding habits are usually more evolutionarily conservative than associations with different host plant taxa (Farrell & Sequeira, 2004; Marvaldi et al., 2002; Morse & Farrell, 2005), however, for other groups of insects it has been shown that shifts in larval feeding habits may occur at a significantly greater rate (Cook et al., 2002; Joy & Crespi, 2007). The study of plant-insect interactions has traditionally focused on host taxa associations (driven by host secondary compounds), however, the use of different plant organs represents another important dimension in the space of available resources that can potentially promote diversification by facilitating species coexistence (Nyman, 2010). Although it has been recognized that larval feeding habits represent ecological traits that may have important macroevolutionary consequences for phytophagous insects (Futuyma & Agrawal, 2009; Nyman, 2010), there are relatively few quantitative studies explicitly assessing their role in diversification processes (Farrell & Sequeira, 2004; Leppänen et al., 2012; Nyman et al., 2006, 2010).

Here, we use a previously inferred phylogeny from mitochondrial and nuclear DNA sequence data (Hernández-Vera et al., 2013) in conjunction with phylogenetic comparative methods (FitzJohn, Maddison & Otto, 2009; Hernández et al., 2013; Pagel, 1999; Pagel, Meade & Barker, 2004) to investigate the evolution of traits of host plant use and their role on the diversification of *Rhinusa* and *Gymnetron* weevils. The specific aims of the study were to: (1) assess phylogenetic conservatism in host use by plant family and in different modes of parasitic behavior; (2) estimate ancestral states of traits associated with host plant use; and (3) assess possible effects of these traits on speciation and extinction rates within the *Rhinusa*/*Gymnetron* species complex. These analyses should provide valuable insights into the evolution of different traits of host plant use and their role in diversification processes of phytophagous insects that feed and develop inside plant tissues.

## Materials and Methods

### Study system data

We used the Bayesian consensus tree and a random sample of 1,000 post burn-in trees generated by Hernández-Vera et al. (2013) from a DNA sequence alignment comprising 3,943 bp of concatenated sequences from two mitochondrial (cytochrome c oxidase subunit II and 16S ribosomal RNA (16S)) and three nuclear gene fragments (elongation factor-1α, arginine kinase and 18S ribosomal RNA (18S)). The sampled taxa include 32 species of *Rhinusa* representing approximately 80% of recognized species, with representatives from the three main taxonomic groups proposed by Caldara, Sassi & Toševski (2010) and 36 species of *Gymnetron* from the Palearctic and Afrotropical regions (approximately 40% of recognized species). Additionally, since some of the analyses performed in this study require ultrametric trees, we performed a Bayesian analysis for the same dataset of concatenated sequences using BEAST 2.1.3 (Bouckaert et al., 2014). For this analysis, two Monte Carlo Markov chains (MCMC) were run for 40 million generations, sampling every 4,000 generations using independent relaxed molecular clock models for the mitochondrial and nuclear gene sequences. Parameterisation of the prior distribution for the mutation rate was as in Hernández-Vera et al. (2013). Convergence of the MCMC chains and posterior probability distributions of the sampled parameters were examined graphically using the program Tracer 1.6 (Rambaut et al., 2014). The obtained maximum clade credibility (MCC) tree and a random sample of 1,000 trees from the posterior probability distribution were used in subsequent analyses. Host plant associations were determined by (a) direct observation of emerging weevils from host plants in the field, (b) records from published literature (Caldara, 2001, 2003, 2008; Caldara, Sassi & Toševski, 2010), and (c) technical reports on studies of host plant use and host preferences in which individuals were reared and monitored through adult emergence (Gassmann & Paetel, 1998; Groppe, 1992; Toševski, Gassmann & Desančić, 2007).

### Assessing phylogenetic conservatism of traits associated with host plant use

We used Mesquite 3.1 (Maddison & Maddison, 2016) to assess phylogenetic conservatism of traits associated with host plant use by testing whether the minimum number of evolutionary steps in a character on a phylogenetic tree is lower than expected by chance (Maddison & Slatkin, 1991). For each trait, a null probability distribution was generated by randomly reshuffling the character data 10,000 times across the terminal taxa of the consensus Bayesian tree. Additionally, we assessed phylogenetic conservatism in a likelihood framework by estimating Pagel’s lambda (λ) parameter (Pagel, 1999) using the fitDiscrete function implemented in the R package GEIGER (Harmon et al., 2008) for discrete characters employing Markov models. This method provides a continuously varying parameter to assess trait variation associated with a phylogeny; a lambda value equal or close to one is indicative of strong phylogenetic signal, that is, the evolution of a trait is not independent of phylogeny, as opposed to values equal or close to zero (Freckleton, Harvey & Pagel, 2002). Statistical significance of the difference between log-likelihood values of models with the estimated lambda and models assuming no phylogenetic signal (λ = 0) was evaluated with likelihood ratio tests (Münkemüller et al., 2012). In both cases, outgroup taxa were excluded and ingroup taxa pruned so that each species was represented by one individual. Host plant use was categorized according to the plant families parasitized by the weevils, namely Plantaginaceae and Scrophulariaceae, as recently circumscribed (Schäferhoff et al., 2010; The Angiosperm Phylogeny Group, 2016). Additionally, host plant use was categorized according to modes of parasitism on different plant structures: (i) roots, (ii) stems and (iii) fruit/seed capsules, and as gall inducers vs. non gall inducers.

### Ancestral state reconstruction of traits associated with host plant use

Using the same host plant use categorization as for phylogenetic conservatism analyses, ancestral states were reconstructed across the random sample of 1,000 post burn-in trees from the analysis performed with MrBayes. We used the Bayesian approach reversible jump MCMC (rjMCMC), implemented in BayesTraits 2.0 (Pagel, Meade & Barker, 2004); the advantage of this method is the possibility to integrate over both uncertainty in the phylogeny and the set of plausible models of trait evolution, rather than conditioning inferences on a specific model (Huelsenbeck, Larget & Alfaro, 2004; Pagel & Meade, 2006). The ancestral states reconstruction was performed for the two most basal nodes of the ingroup taxa (labelled as Nodes 1 and 2) using the command “AddMRCA,” which identifies the most recent common ancestor to a group of species and reconstructs the state of that node, combining information across trees (Pagel, Meade & Barker, 2004). Based on preliminary maximum likelihood analyses, we employed an exponential hyper-prior with mean values drawn from a uniform distribution with an interval 0–10. A total of 100 million iterations were run discarding the first 25% as burn-in, sampling the Markov chain every 37,500 iterations. To assess convergence of the Markov chains, analyses were run at least twice and posterior distributions of the estimated parameters were examined in Tracer 1.6 (Rambaut et al., 2014). Posterior probability density plots were generated as violin plots (Hintze & Nelson, 1998) with the R package Vioplot 0.2 (Adler, 2005).

For comparison with the rjMCMC approach and to account for differential speciation, extinction and character transition rates in the reconstruction of ancestral states, we additionally performed ancestral state reconstructions using maximum likelihood under binary and multi-state speciation and extinction models (BiSSE/MuSSE) (FitzJohn, 2012; Maddison, Midford & Otto, 2007) in the R package Diversitree 0.9-6. Analyses were performed using the MCC tree obtained with BEAST and the models of trait evolution with the highest posterior probability according to the results of the rjMCMC analyses.

### Assessing effects of traits associated with host plant use on speciation and extinction rates

A BiSSE model was employed to assess the effect of binary traits on speciation and extinction rates within the *Rhinusa*/*Gymnetron* species complex. We independently used the model to assess the effect of host plant family use (Scrophulariaceae or Plantaginaceae) and the mode of parasitism as gall or non-gall inducer. The model estimates six parameters; speciation (λ) and extinction (μ) rates for states 0 and 1 (λ0, λ1, μ0, μ1) and transition rates from state 0 to 1 and vice versa (q01 and q10). Similarly, a multi-state speciation and extinction (MuSSE) model was employed to assess the effect of different modes of parasitism on either roots, stems or fruit/seed capsules. The MuSSE model is an extension of the BiSSE model to discrete traits with more than two states or combinations of binary traits analyzed simultaneously (FitzJohn, 2012). Models with asymmetrical state-dependent speciation and extinction rates were compared against models with speciation and extinction rate parameters constrained to be equal for all states. Parameter values for both unconstrained and constrained models were estimated with the R package Diversitree 0.9-6 (FitzJohn, 2012) for the sample of 1,000 post burn-in trees from the Bayesian analysis with BEAST, thus taking into account phylogenetic uncertainty. We corrected for incomplete sampling (FitzJohn, Maddison & Otto, 2009) by specifying the proportion of species included in the phylogeny (0.52) with the argument “sampling.f.” Statistical significance of model differences was assessed by performing likelihood ratio tests as implemented in Diversitree. To graphically visualize net diversification rates (λ—μ) associated with the different traits of host plant use, posterior density plots were generated with Bayesian analyses performed in Diversitree by running 10,000 Markov chains using the MCC tree and the same BiSSE and MuSSE models as above. For comparison with the Bayesian estimates and to account for phylogenetic uncertainty, diversification rates were also estimated with maximum likelihood analyses from 1,000 post burn-in trees inferred with BEAST and the mean values were plotted on the posterior density plots.

## Results

### Phylogenetic conservatism of traits associated with host plant use

*Rhinusa* and *Gymnetron* represent two non-reciprocally monophyletic groups with deep genetic divergences between southern Africa and Palearctic lineages (Hernández-Vera et al., 2013). Results from both parsimony and likelihood-based tests indicate that there is statistically significant phylogenetic conservatism in host use by plant family and modes of parasitism. Characters of host use by plant family are not randomly distributed across the Bayesian consensus tree (*p* < 0.001). Eight parsimony steps were observed against 14 expected steps at the lower confidence limit under 10,000 character randomizations of the null model. When host plant use is categorized according to the plant parts being parasitized and as gall inducer vs. non gall inducer, the distribution of characters over the Bayesian consensus tree is marginally significant at the 0.05 confidence level, that is, in both cases the difference between the expected (null model) and observed number of parsimony steps is only one. A similar trend was observed for the results from the estimation of Pagel’s lambda parameter, which indicate a strong phylogenetic signal for host use by plant family (λ = 1) but less phylogenetic signal for both host use as either gall or non-gall inducer and parasitic mode on different plant structures (Table 1).

**Table 1:** Summary statistics of Pagel’s lambda parameter estimated from the MCC tree inferred with BEAST and different categorization schemes of host plant use.

	Estimated lambda value	Log-likelihood of models		
		lambda set to zero	lambda estimated	χ^2^ value (d*f* = 1)
Parasitism on either roots, stems or fruit and seed capsules	0.772	−45.588	−41.198	8.782**
Gall vs. non gall inducers	0.927	−26.591	−22.461	8.261**
Host use by plant family	1.0	−32.462	−13.398	38.128***

**Notes:**

Lambda values equal or close to one indicate strong phylogenetic signal. Log-likelihood values from the estimates of lambda were contrasted with those obtained from models where lambda was set to zero (no phylogenetic signal). Likelihood ratio tests were approximated with a Chi-squared (χ^2^) distribution.

** *p*-value < 0.01.

*** *p*-value < 0.001.

### Reconstruction of ancestral character states

**Figure 1:**
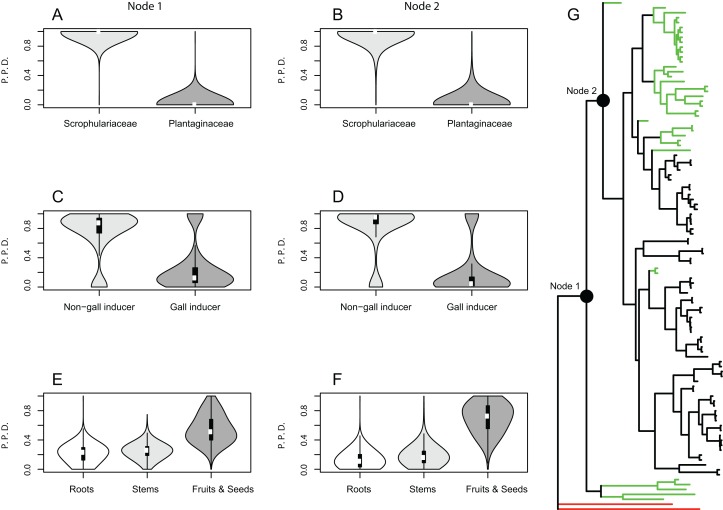
Bayesian ancestral state reconstructions for different traits of host plant use by *Rhinusa* and *Gymnetron* weevils. Violin plots show posterior probability densities (P.P.D.) of ancestral states for different traits of host plant use by *Rhinusa* and *Gymnetron* species: (A–B) use of either Scrophulariaceae or Plantaginaceae taxa as hosts, (C–D) gall vs. non-gall inducer behavior, and (E–F) parasitic behavior on three different plant organs. White dots and vertical bars inside density plots represent the median and interquartile range, respectively. Using rjMCMC analyses and the “AddMRCA” command in BayesTraits, reconstructions were performed across a random sample of 1,000 post burn-in trees for the two most basal nodes labelled as 1 and 2 in the majority-rule consensus tree obtained with MrBayes (G). Branch colors indicate the genera and outgrup taxa; green = *Gymnetron*, black = *Rhinusa*, red = outgroup.

Results from the Bayesian analyses provide strong support for the family Scrophulariaceae and non-gall inducing parasitic behavior as ancestral character states of host plant use by *Gymnetron* and *Rhinusa*. The estimated mean posterior probability for Scrophulariaceae as the ancestral state at the two most basal nodes 1 and 2 was 0.99 and 0.96, respectively, whereas for non-gall inducing behavior the values were 0.75 and 0.81 for the same nodes. A parasitic behavior on fruits and seed capsules as the ancestral condition appears weakly supported as the mean posterior probability values were 0.55 and 0.69 at nodes 1 and 2, respectively (Fig. 1). A single-rate model of trait evolution was the most frequently sampled model by the rjMCMC for each of the three schemes of host plant use, but with different restrictions on the transition rates. For use of host plant family the transition rate from Plantaginaceae to Scrophulariaceae was equal to zero, for gall vs non-gall parasitic behavior both transition rates were equal, and for parasitic behavior on three different plant organs all transition rates were equal except for the transition from fruit and seed capsules to roots, which was equal to zero.

**Figure 2:**
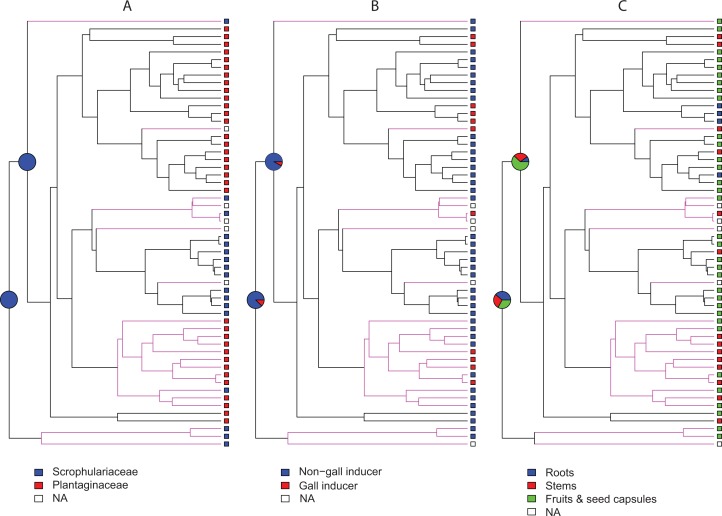
Maximum likelihood ancestral state reconstructions. Employing the MCC tree generated with BEAST, ancestral state reconstructions were performed under BiSSE and MuSSE models for different traits of host plant use by *Rhinusa* (black branches) and *Gymnetron* (magenta branches) species: (A) use of Scrophulariaceae or Plantaginaceae as hosts, (B) gall vs. non-gall inducer behavior, and (C) parasitic behavior on three different plant organs. Pie graphs represent proportional likelihoods of alternative character states; NA stands for not available trait data.

Ancestral state reconstructions under BiSSE and MuSSE models recovered the same ancestral states as rjMCMC analyses for all host plant use traits, except for parasitic behavior on fruits and seed capsules as the ancestral state at node 1. In this case the result is equivocal with a proportional likelihood value of 0.33, hence the three states are equally probable. For node 2, parasitic behavior on fruits and seed capsules as the ancestral state is weakly supported with a proportional likelihood value of 0.55. Proportional likelihoods for use of Scrophulariaceae as the ancestral condition in nodes 1 and 2 are 1.0 in both cases, whereas the values for non-gall inducer behavior as the ancestral state are 0.88 and 0.92 for nodes 1 and 2, respectively (Fig. 2).

### Effects of traits associated with host plant use on speciation and extinction rates

**Figure 3:**
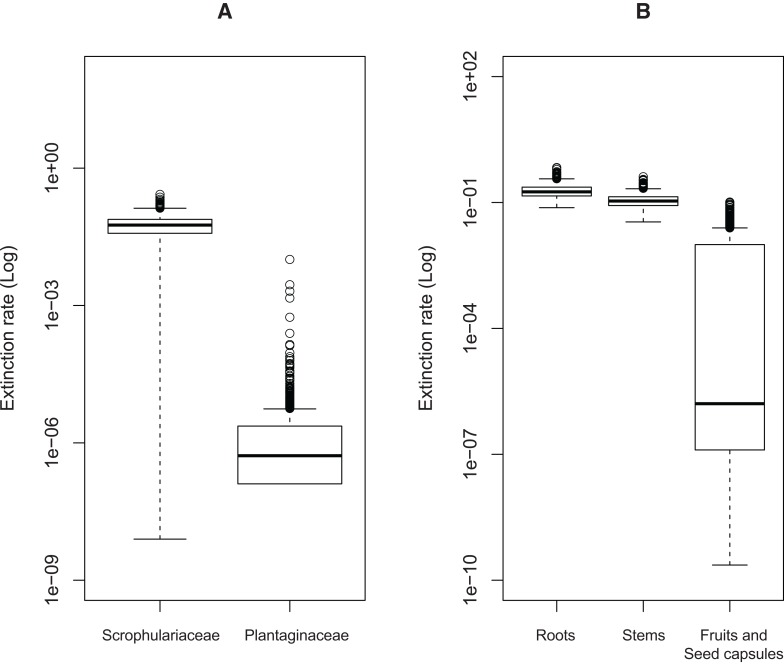
Estimated extinction rates associated with different traits of host plant use by *Rhinusa* and *Gymnetron* weevils. (A) Host plant use categorization according to plant family. (B) Host plant use categorization according to parasitic behavior on different plant organs. Rates were estimated under BiSSE and MuSSE models (FitzJohn, 2012) from 1,000 post burn-in Bayesian trees.

**Figure 4:**
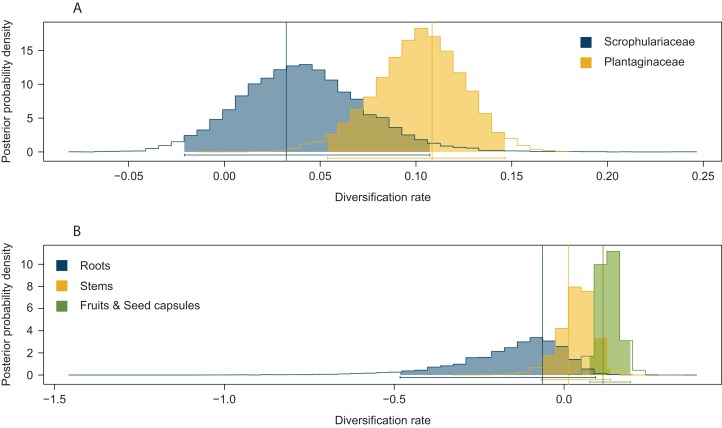
Posterior probability density plots of net diversification rates (λ—μ) for *Rhinusa* and *Gymnetron* weevils associated with two traits of host plant use. (A) Use of two different plant families as hosts, (B) parasitic behavior on three different plant organs. Parameters were estimated from the MCC tree generated with BEAST under BiSSE and MuSSE models, respectively. Vertical lines represent mean maximum likelihood estimates from 1,000 post burn-in trees inferred with BEAST.

Significant effects of traits associated with host plant use on extinction rates were found in two cases. The symmetric model assuming equal extinction rates for the use of Plantaginaceae or Scrophulariaceae as hosts was rejected in 728 of the 1,000 likelihood ratio tests performed (Fig. S1A). Likewise, 790 out of 1,000 likelihood ratio tests rejected the symmetric model assuming equal extinction rates for weevils parasitizing either roots, stems or fruits and seed capsules (Fig. S1B). Extinction rates associated with use of plants within Plantaginaceae as hosts were consistently estimated to be equal or close to zero for the 1,000 analyzed post burn-in trees (Fig. 3A); similarly, the lowest extinction rates were estimated for weevils parasitizing fruits and seeds (Fig. 3B). Because low sample size (56 tips in our tree) may reduce the accuracy and precision of parameter estimation in trait-dependent diversification analyses (Davis, Midford & Maddison, 2013), we additionally simulated fully sampled trees using the previously estimated speciation and extinction rates (median values from the ML analyses performed on 1,000 post burn-in trees) and then we inferred those rates after pruning the trees to the proportion of taxa in our original tree (0.52). The results show that both BiSSE and MuSSE analyses are able to correctly estimate extinction rates from the pruned tree (Fig. S2). No evidence of character state-dependent speciation rates was found for any of the traits associated with host plant use. Bayesian estimation of net diversification rates (λ—μ) associated with use of host plant family and parasitic behavior on different plant parts are presented as density plots in Fig. 4. Consistent with the likelihood estimates of low extinction rates, higher diversification rates were estimated for weevils using Plantaginaceae taxa as hosts and parasitizing fruits and seed capsules.

## Discussion

### Phylogenetic conservatism of host plant use

Our results provide strong evidence for phylogenetic conservatism of host use at the plant family level. Although examples of generalist plant-feeding insects exist (Ali & Agrawal, 2012; Bernays & Minkenberg, 1997; Forister et al., 2015; Ribeiro et al., 2005), the most commonly observed pattern is that most species are restricted to a few closely related plant taxa where related insects tend to feed on related groups of plants, that is, host plant use is maintained over evolutionary time scales (Jaenike, 1990; Janz & Nylin, 1998; Winkler & Mitter, 2008). Since the publication of Ehrlich & Raven’s (1964) influential paper, it has been recognized that host plant chemistry (secondary compounds) and insect dietary tolerances play a significant role in shaping insect-host plant associations (Becerra, 1997; Berenbaum, 1983; Feeny, 1975; Nishida, 2014; Wahlberg, 2001). At high taxonomic ranks such as plant family, plants may exhibit important differences in their secondary compounds, making host shifts less frequent due to the difficulty of insects to metabolize different compounds (Becerra, 1997; Futuyma & McCafferty, 1990; Janz & Nylin, 1998).

In the case of the host plant families utilized by *Rhinusa* and *Gymnetron*, there is an apparent pattern of mutually exclusive occurrence of two types of iridoid glycosides (Boros & Stermitz, 1990; Bowers, 1991; Jensen, Nielsen & Dahlgren, 1975). Host plants in the Scrophulariaceae synthetize aucubine and or catalpol, whereas host plants within Plantaginaceae lack of these compounds or they are present in only small amounts. Except for the genus *Veronica*, the main iridoid constituents of hosts within Plantaginaceae are antirrhinoside, antirrhinum glycoside B, and or asarina glycoside (Kooiman, 1970). Hence, qualitative and quantitative variation in the levels of these compounds might explain, at least in part, host use at the plant family level. It has been shown that some species included in the same tribe of our focal group (Mecinini) and the closely related Cionini, exhibit metabolic differences in the sequestration of these compounds. Weevils of the genera *Cionus* and *Cleopus* sequester iridoid glycosides from their host plants *Scrophularia* and *Verbascum*, in contrast, there is no evidence of sequestration of these compounds by weevils of the genera *Mecinus* and *Rhinusa* (Baden, Franke & Dobler, 2012; Baden, Franke & Dobler, 2013; Jamieson & Bowers, 2010). Although iridoid glycosides may play a significant role in the interaction of *Gymnetron* and *Rhinusa* weevils with their hosts, perhaps as feeding stimulants and oviposition cues (Nieminen et al., 2003; Reudler Talsma et al., 2008), further studies will be necessary to elucidate the exact mechanism of host plant preferences, as concentrations of secondary metabolites may be affected by a variety of abiotic and biotic factors (Jamieson & Bowers, 2010, 2012).

The lower phylogenetic signal for traits of parasitic behavior suggest they are more labile ecological traits. This finding contrasts with evidence reported for several beetle groups that suggests larval feeding habits and modes of parasitism exhibit higher phylogenetic conservatism than host plant taxa associations (Farrell & Sequeira, 2004; Marvaldi et al., 2002; Morse & Farrell, 2005). Other studies on gall-inducing insects have found evidence that shifts between host plant organs can occur at a significantly greater rate than shifts between different host taxa (Cook et al., 2002; Joy & Crespi, 2007). Modes of parasitism on different plant structures may represent alternative ecological axes for niche expansion and a finer partitioning of resources, as they can reduce potential inter- and intraspecific competition (Denno, McClure & Ott, 1995; Klomp, 1964; Price, 1980). Mediated by marking pheromones, many parasitoids and phytophagous insects avoid hosts already infested by conspecifics or closely related species (Anderson, 2003; Nufio & Papaj, 2001; Roitberg & Prokopy, 1987). In beetles, there is evidence that ovipositing females of some species within the families Chrysomelidae (Guedes & Yack, 2016; Messina & Renwick, 1985) and Curculionidae (Addesso et al., 2007; Ferguson et al., 1999) avoid hosts and plant structures that have been previously utilized by other females for laying eggs. Hence, driven by chemical constraints, the use of a restricted set of host plants over evolutionary time scales may lead to inter- and intraspecific competition which in turn could facilitate an escape-and-radiate scenario (Ehrlich & Raven, 1964) where weevils “escape” to different plant structures, potentially undergoing diversification. An example of this is illustrated by five species of *Rhinusa* exploiting different resources within the same host plant species *Linaria vulgaris. R. antirrhini* feeds and develops inside fruit capsules, whereas *R. linariae* and *R. pilosa* are both gall inducers, the former utilizes roots and the latter stems. A further level of ecological resource partitioning is present with two other species, *R. collina* and *R. eversmanni*, acting as inquilines of the galls induced by *R. linariae* and *R. pilosa,* respectively (Gassmann et al., 2014; Toševski, Gassmann & Desančić, 2007).

### Evolution of host plant use and diversification

Results from Bayesian and maximum likelihood methods provide strong support for use of host plants within Scrophulariaceae as the ancestral condition for *Rhinusa* and *Gymnetron*. This is consistent with the hypothesis put forward by Hernández-Vera et al. (2013) for a South African origin for this species complex, given the predominant concentration of Scrophulariaceae genera in the southern hemisphere, particularly Africa (Olmstead et al., 2001; Tank et al., 2006). Thus, weevils initially specialized on plants within Scrophulariaceae and eventually colonized Plantaginaceae, a new set of plants closely related to the ancestral ones (Albach, Meudt & Oxelman, 2005). Results from the rjMCMC analyses also support this hypothesis, as the most frequently sampled model of trait evolution was a single-rate model with transition rates from Plantaginaceae to Scrophulariaceae restricted to zero. The estimated higher diversification rates associated with use of Plantaginaceae taxa as hosts (Fig. 4) suggest that this colonization event facilitated the diversification of the group, exploiting and adapting to newly-opened ecological niche space, potentially reducing competition for resources and providing an enemy-free space (Murphy, 2004; Schluter, 2000). It is worth noting that most of the taxa (five out of six) utilized as hosts within Plantaginaceae are included in the tribe Antirrhineae, a group of plants characterized for synthesizing antirrhinoside, an iridoid glycoside that appears to function as a deterrent for some generalist herbivores but also as an attractant for others, particularly those exhibiting a more specialized diet (Beninger, Cloutier & Grodzinski, 2008; Bowers, 1991). Thus, the unique secondary compound present in the alternative host may have represented an advantage over the ancestral host taxa.

A non-gall inducing behavior is also supported as the ancestral condition for *Rhinusa* and *Gymnetron* weevils. Based on the scarcity or absence of gall inducers in the most ancestral families within Curculionoidea, it has been suggested that galling represents a derived specialized life history in weevils (Korotyaev et al., 2005). Galls may represent an expansion of ecological resources for *Rhinusa* and *Gymnetron*, providing enhanced nutrition and a favorable microhabitat that facilitates their larval development (Price, Fernandes & Waring, 1987; Stone & Schönrogge, 2003). However, we did not find evidence of any effect of either gall or non-gall inducing behavior on speciation and extinction rates. Although gall-inducing insect groups are considered to be more host-specific than their non-galling relatives (Hardy & Cook, 2010; Price, Fernandes & Waring, 1987; Shorthouse & Rohfritsch, 1992), to date there is no conclusive evidence of increased diversification rates in gallers. The effect of gall inducing behavior on net diversification rate appears to be lineage specific, where host range may also play an important role (Hardy & Cook, 2010).

The ancestral state reconstruction of parasitic behavior on different plant structures is highly uncertain, and as a consequence it is difficult to infer the plesiomorphic condition. It has been reported that larvae of *Gymnetron*, *Rhinusa* and the closely related genus *Mecinus* do not exhibit a general pattern regarding the direction of evolution in their modes of parasitism (Caldara, Sassi & Montagna, 2013; Caldara, Sassi & Toševski, 2010). This apparent lack of directionality is consistent with our results from phylogenetic conservatism analyses supporting the parasitic behavior as a labile ecological trait. On the other hand, results from the trait-dependent diversification analyses under the MuSSE model suggest that endoparasitism of fruits and seed capsules has contributed to the diversification of *Rhinusa* and *Gymnetron* weevils (Figs. 3B and 4). The use of these reproductive structures may represent an advantage, as they provide higher amounts of nutrients for the developing endophagous larvae (Hulme & Benkman, 2002; Janzen, 1971) and a favorable micro-environment which may help to overcome the problem of desiccation of the immature stages (Anderson, 1993). Additionally, seeds can contain high concentrations of secondary compounds toxic to some vertebrates, insects or microbes and thus provide protection to certain specialist seed predators (Janzen, 1971). It has been shown that concentrations of antirrhinoside and other iridoid glycosides are usually high in reproductive organs in some host species utilized by *Rhinusa* and *Gymnetron* in the tribe Antirrhineae (Beninger et al., 2007; Jamieson & Bowers, 2010).

Interestingly, our results indicate that the enhanced diversification rates associated with the use of Plantaginaceae and endoparasitism of fruits and seed capsules, are driven by low extinction rates (Figs. 3A–3B). Traditionally, studies have focused on the mechanisms promoting speciation in Coleoptera, particularly plant-feeding beetles (Farrell, 1998; McKenna et al., 2009; Mitter, Farrell & Wiegmann, 1988), however, it may also be fruitful to pay attention to the factors that have inhibited their extinction (Smith & Marcot, 2015). Although extinction estimates from phylogenies of present day species have been called into question (Quental & Marshall, 2010; Rabosky, 2010), of relevance is the fact that all living weevil families are known from the fossil record, and all weevil families in the fossil record are extant (Gratshev & Zherikhin, 2003; McKenna et al., 2009). This is consistent with the evidence that reduced extinction has played an important role in the extraordinary diversity of modern insects (Condamine, Clapham & Kergoat, 2016; Labandeira & Sepkoski, 1993) and particularly beetles (Hunt et al., 2007; McKenna et al., 2015).

## Conclusions

*Rhinusa* and *Gymnetron* weevils initially specialized on plants within Scrophulariaceae and then shifted to the closely related Plantaginaceae. Likewise, their gall inducing behavior represents a derived specialized trait which evolved from non-galler weevils possibly in response to resource competition given that usually more than one species utilize the same host (although other explanations are possible, such as escape from parasites/predators), providing enhanced nutrition and a favorable microhabitat which facilitates larval development. The use of restricted sets of host plants is phylogenetically conserved, most likely because of substantial differences in the chemical composition of their hosts, thus reducing the probability of host shifts. In contrast, the utilization of different plant organs represents a more labile ecological trait that allows a finer partitioning of resources; this ecological niche expansion is associated with enhanced diversification rates in weevils exhibiting a parasitic behavior on fruits and seed capsules. Our results show that ecological factors such as host plant use and specialized endoparasitic habits can promote diversification via low extinction rates in phytophagous insects that require plant tissues for the completion of their reproductive cycle.

## Supplemental Information

10.7717/peerj.6625/supp-1 Supplemental Information 1 Histograms of *p*-values (α = 0.1) from 1000 likelihood ratio tests comparing symmetric (equal rates, null model) versus asymmetric (different rates) models of extinction rates. Extinction rates associated with (A) use of either Scrophulariaceae or Plantaginaceae as host plant families and (B) use of different plant organs: roots, stems or fruits and seed capsules.

10.7717/peerj.6625/supp-2 Supplemental Information 2 Posterior probability density plots of extinction rates. Extinction rates associated with use of Plantaginaceae taxa as hosts (A) and use of fruits and seed capsules (B) estimated with MCMC analyses (BiSSE and MuSSE models) from pruned simulated trees. The red line indicates the true parameter value used to simulate the tree.

## Additional Information and Declarations

### Data Availability

The following information was supplied regarding data availability: Original dataset of DNA sequences is in https://www.treebase.org/, Study ID S13611. Hernández-Vera, Gerardo (2019): Phylogenetic Trees & Data. figshare. Fileset. DOI 10.6084/m9.figshare.7117928.v2. Hernández-Vera, Gerardo (2018): Simulations of phylogenetic trees under BISSE and MUSSE models and MCMC analyses. figshare. Code. DOI 10.6084/m9.figshare.7117988.v1.
