# Supplementary figures and images for: Evolution of host plant use and diversification in a species complex of parasitic weevils (Coleoptera: Curculionidae)
Source: PeerJ. 2019 Mar 20;7:e6625. doi: 10.7717/peerj.6625 (PMC6431137; doi:10.7717/peerj.6625)

**A**

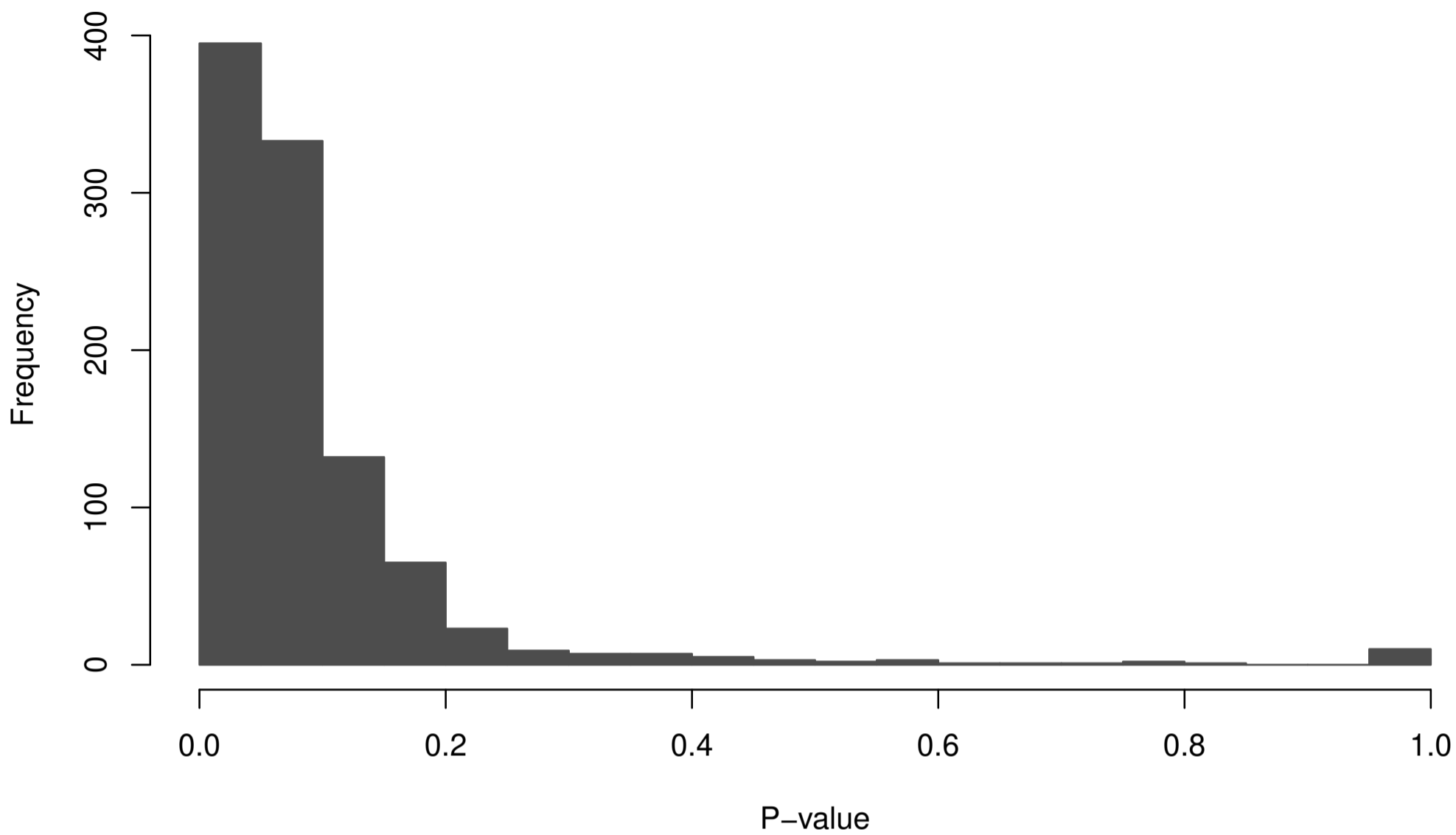

**B**

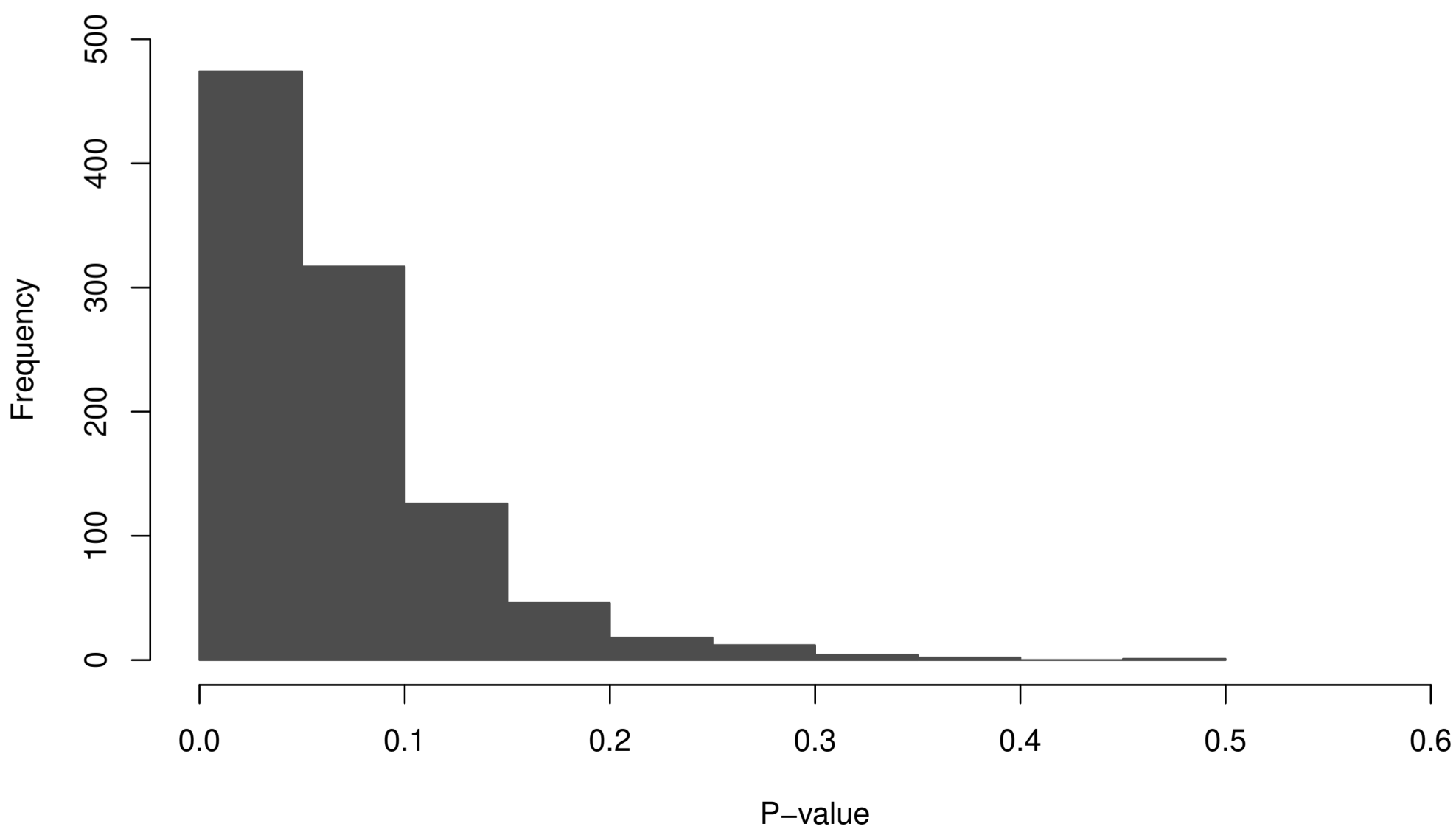

Supplement: Supplemental Information 1 — Extinction rates associated with (A) use of either Scrophulariaceae or Plantaginaceae as host plant families and (B) use of different plant organs: roots, stems or fruits and seed capsules. [file peerj-07-6625-s001.pdf]

A

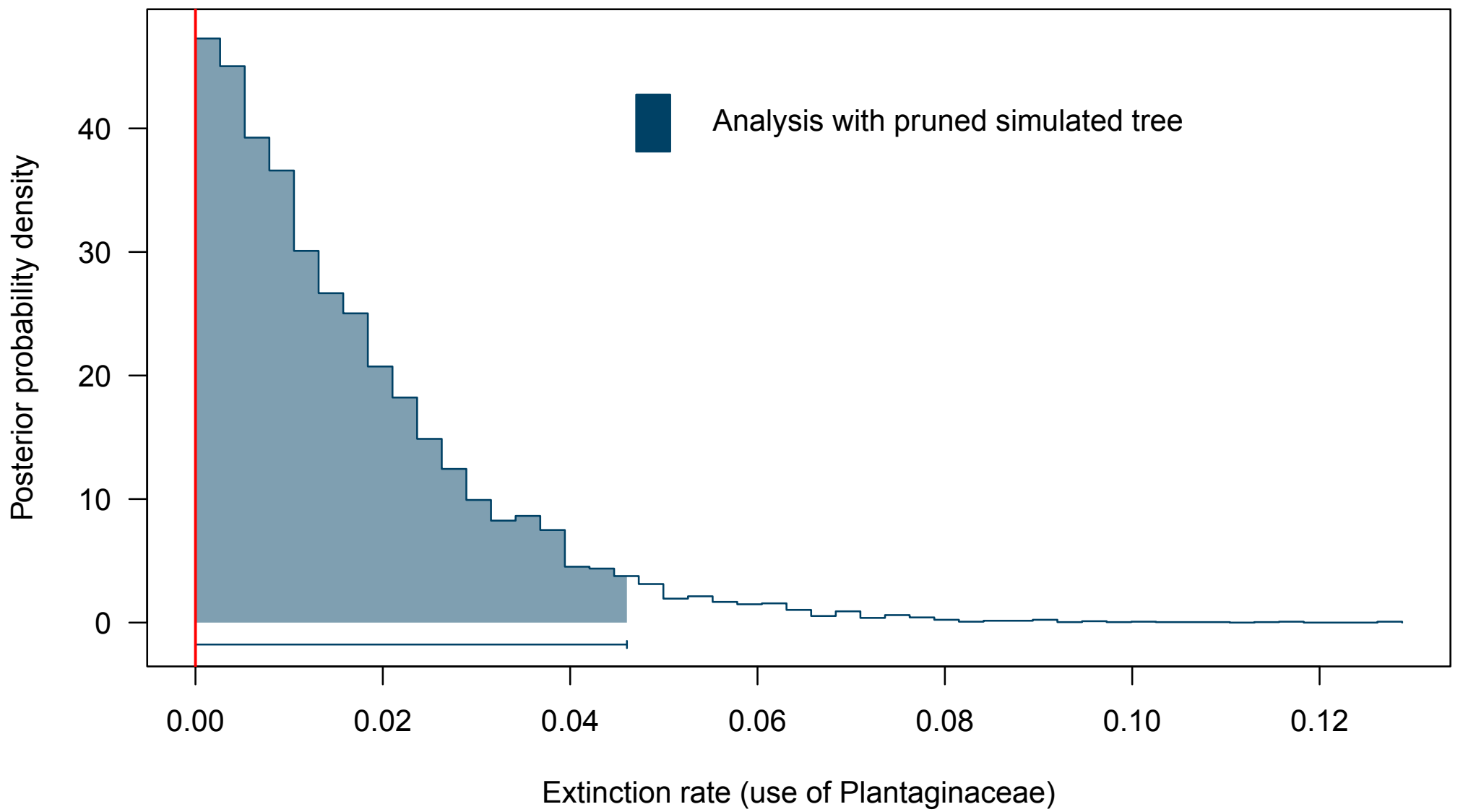

B

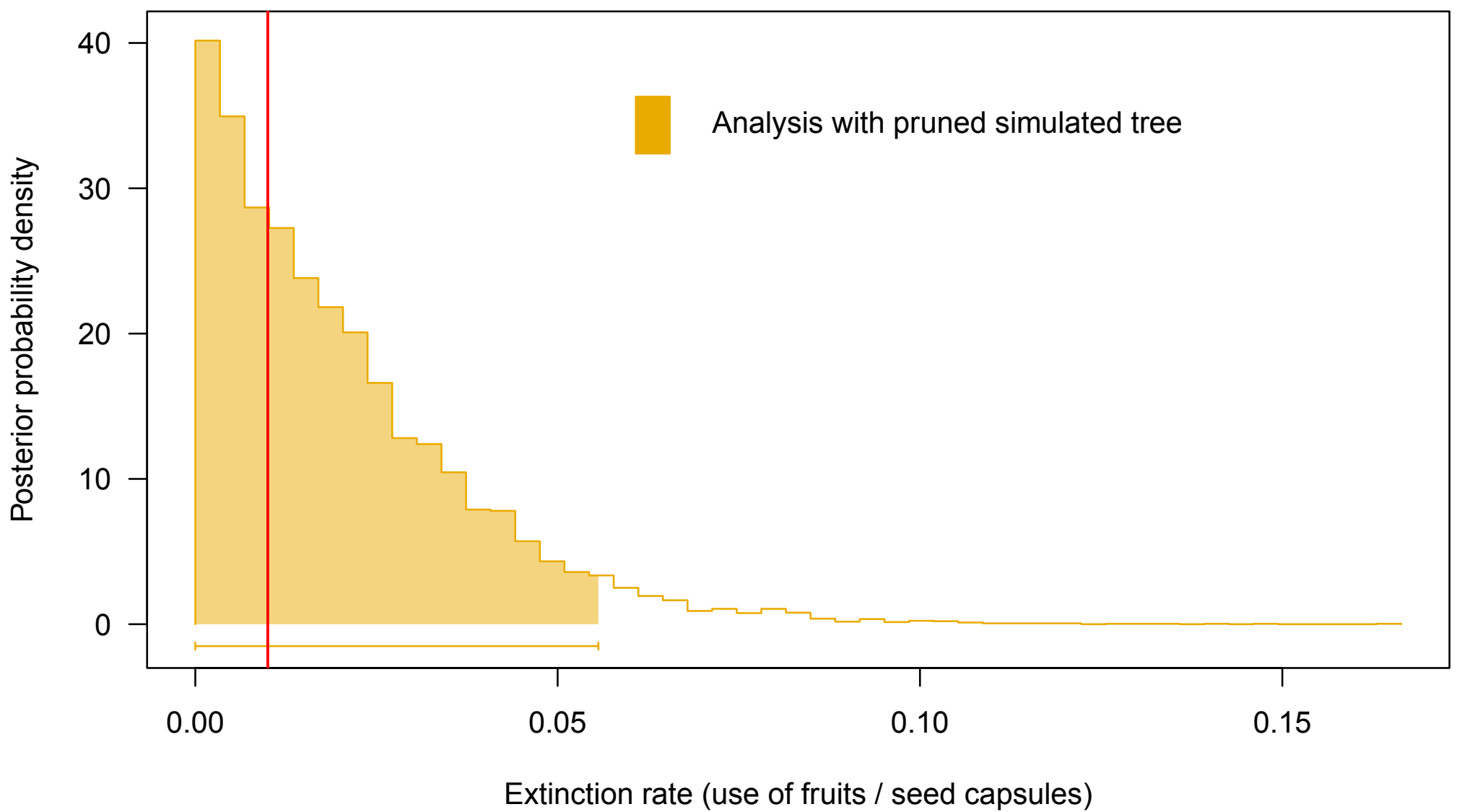

Supplement: Supplemental Information 2 — Extinction rates associated with use of Plantaginaceae taxa as hosts (A) and use of fruits and seed capsules (B) estimated with MCMC analyses (BiSSE and MuSSE models) from pruned simulated trees. The red line indicates the true parameter value used to simulate the tree. [file peerj-07-6625-s002.pdf]
